# P05.17. Addressing nature deficit disorder: a mixed methods study of social well-being among young adults attending a wilderness science camp

**DOI:** 10.1186/1472-6882-12-S1-P377

**Published:** 2012-06-12

**Authors:** S Warber, M Marselle, A Dehudy, K Irvine

**Affiliations:** 1 University of Michigan, Ann Arbor, USA; 2 De Montfort University, Leicester, United Kingdom

## Purpose

An increasingly ‘screen-based’ culture raises concerns for the health and well-being of America’s youth. We investigated whether nature-based experiences affected the social well-being of young adults attending a three-week wilderness camp.

## Methods

Online surveys (pre, post) were administered. We used paired t-tests (N=35) to analyze differences in social well-being as measured by Ryff’s Positive Relations with Others subscale. In situ interviews (n=20) explored relationships between the camp experience, nature, and well-being. Interviewees were selected to produce a maximum variation sample along dimensions of gender, previous nature experiences, and nature connectedness. Analysis of interviews identified elements of social connection and the role played by nature.

## Results

Quantitative assessment of social well-being showed no significant change (74.17+/-12.39 vs. 77.40+/-13.28, p=0.066). Interview results, however, strongly emphasized social relationships describing the process of making friends, the importance of being part of a group, and how the wilderness environment facilitated connection. Making friends was enhanced by spending time together, listening to each other, and developing intimacy. Being part of a group included subthemes of a sense of community, engaging in group play, working together as a team, and the bonding effect of shared experiences. The natural environment specifically enhanced these processes through loss of ego, breaking down barriers, and limited distractions. This facilitated deeper relationships than would occur in urban surroundings, and afforded social-relatedness (i.e., acceptance and help), positive feelings (i.e., enjoyment, reward, and love), and a sense of interdependence with others (i.e. learning/teaching, having an impact, and making long-term commitments).

## Conclusion

Nature experiences do enhance social well-being among young adults, but this construct is poorly measured by existing instruments. Further research into the effects of nature-based therapies on young people’s well-being is important for society and requires careful selection or development of measures of the social dimension as actually experienced by participants.

